# The Family Talk Programme in Ireland: A Qualitative Analysis of the Experiences of Families With Parental Mental Illness

**DOI:** 10.3389/fpsyt.2021.783189

**Published:** 2021-11-15

**Authors:** Christine Mulligan, Mairead Furlong, Sharon McGarr, Siobhan O'Connor, Sinead McGilloway

**Affiliations:** Centre for Mental Health and Community Research, Maynooth University, Maynooth, Ireland

**Keywords:** children, COPMI, Family Talk, mental health, mental disorder, mental illness, parents, qualitative research

## Abstract

**Background:** Parental mental illness is common, costly, can lead to children developing mental disorders and impaired lifetime outcomes, and places a substantial burden on caregiving partners. Family Talk (FT) is a widely implemented, 7-session, whole-family programme, with promising evidence of effectiveness in targeting the intergenerational transmission of mental illness. However, to date, very little qualitative research of family experiences of FT has been undertaken. The objectives of this study were to: (1) investigate the experiences of families attending FT; and (2) explore the key facilitators and barriers to engagement in mainstream mental health settings.

**Methods:** This study was nested within a randomised controlled trial (RCT) of Family Talk [*N* = 86 families (139 parents, 221 children)] implemented in 15 adult, child and primary care mental health sites in Ireland. Semi-structured interviews were conducted with a purposive sample of 45 participants, including 23 parents with mental illness (PMI), 7 partners and 15 children/young people aged 9 to 18 years. Interview data were transcribed verbatim and analysed using constructivist grounded theory.

**Results:** Over two thirds of families across sites reported substantial benefits from participation in FT, including reduced stigma, giving children and partners a voice, increased service-user confidence, and improved family communication/relationships. Key facilitators identified by families included: programme delivery by a competent, non-judgmental clinician; the whole-family approach; and family readiness to engage. Barriers to engagement included stigma, family crises/relapse, service constraints, impact of COVID-19, and a need for further child, family and follow-up sessions/supports.

**Conclusion:** This study is the first qualitative analysis of family experiences of FT to be conducted within the context of an RCT and national programme to introduce family-focused practise for families with PMI. The findings illustrate that FT is beneficial across cultural/policy contexts, different mental disorders and can be implemented across adult and child mental health settings, including children with existing mental health challenges. Key barriers and facilitators to implementation were identified by families, all of which should help to inform the future implementation of FT, and other similar interventions, both in Ireland and elsewhere.

## Introduction

It is estimated that 23% of all families have at least one parent who has, or had, a mental illness; this has been shown to increase the risk of children developing a mental disorder during their lifetime (range 41 to 77%), whilst multiplying five-fold their utilisation of health and social services, and placing a substantial emotional, financial and parenting burden on caregiving partners (1–3). In the Republic of Ireland (RoI), 20% of adults experience a mental health illness—the third highest incidence across 36 countries in Europe—costing the Irish state €11 billion per year (4). Furthermore, it is estimated that 280,000 children in the RoI are dependent on parents who have a mental illness (5).

The transmission of risk from parents to children involves a complex interplay of genetic, prenatal, family and environmental/social influences and is significantly mediated by the impact of parental symptoms on parent-child interactions (e.g., insensitive and erratic attunement)(2). Worryingly, these vulnerable families are often not identified or supported by mental health professionals in the RoI, or in other jurisdictions, due to: a lack of policy/practise guidance; little or no collaboration between Adult Mental Health Services (AMHS) and Child and Adolescent Mental Health Services (CAMHS); an individualised, crisis-oriented approach to assessment/treatment; competency and confidentiality concerns amongst mental health professionals who may feel ill-equipped to undertake family work; and parental stigma/fear of social services and losing custody of their children (6, 7).

Although the prevalence and burden of parental mental illness (PMI) is a cause for public concern, there is increasing evidence that integrated prevention and early intervention family-focused programmes/practise (FFPs) can help decrease the risk of developing mental disorders for children by up to 40% (8) and reduce referrals to child protection services (9). The Family Talk programme, in particular, has been identified in several systematic reviews (8, 10, 11) as a key intervention with promising evidence of effectiveness in improving parent and child understanding of mental illness and child internalising symptoms (12–16), with one study indicating enhanced family functioning and parental mental health recovery 4.5 years later (14).

**Figure 1:**
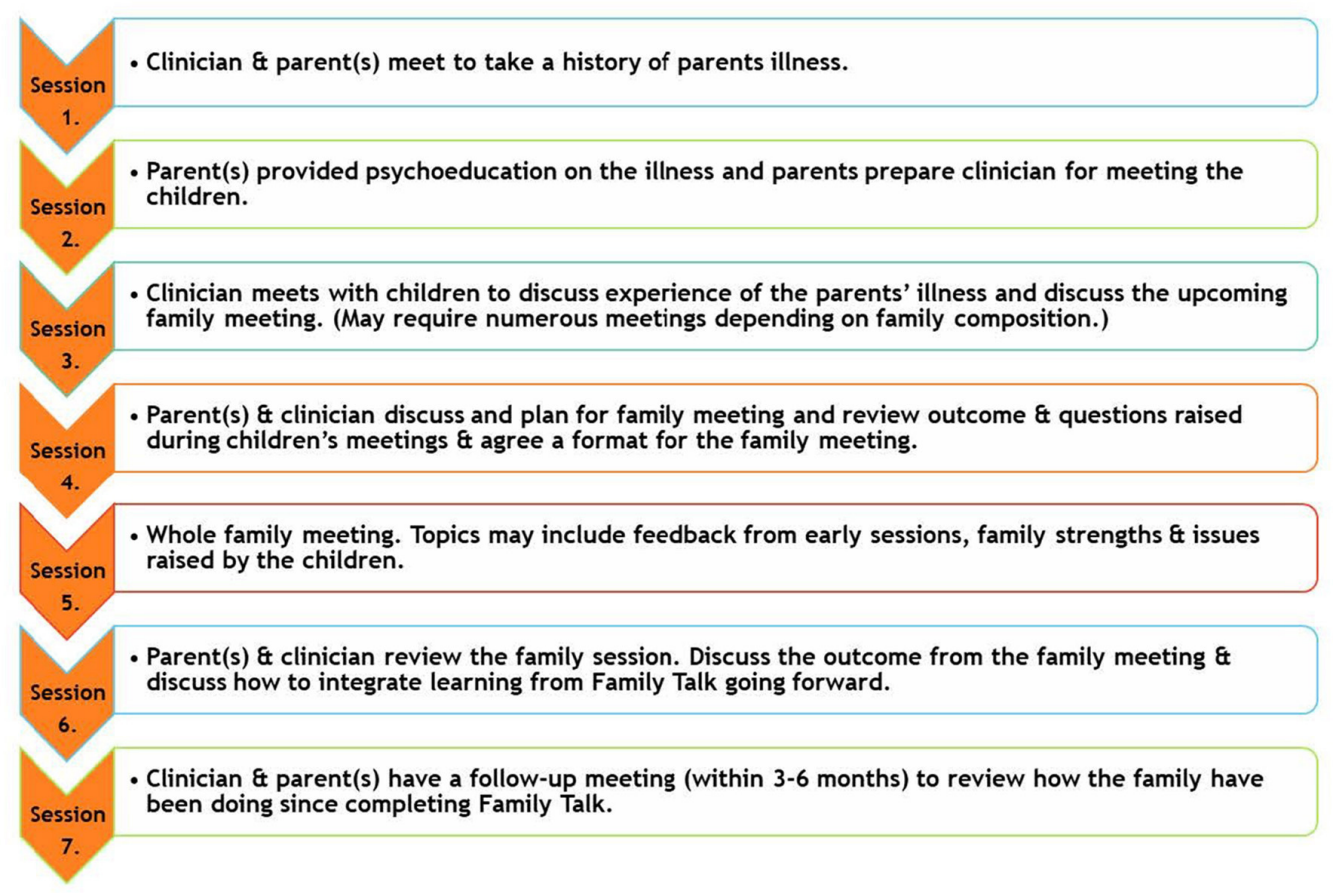
Family Talk sessions.

Family Talk (FT) was developed by William Beardslee and colleagues in the USA in the 1980's and is a manualised, 7-session, strengths-based, psycho-educational, whole-family approach designed to enhance family understanding and communication about parental mental illness, improve family interpersonal relationships, and promote family resilience and utilisation of social supports (12). The intervention involves a clinician meeting with each individual family, i.e. with parents (sessions 1, 2, 4, 6, 7), with each child individually (session 3), and with the whole family (session 4) (see Figure 1). Sessions typically last 60–90 min. The current evidence base for FT is limited by the small number of RCTs conducted to date and within only three countries (USA, Finland, Germany), generally small sample sizes, and mixed support for effectiveness in improving child externalising symptoms, parental mental health and family functioning (14, 15, 17, 18).

Due to its small but growing evidence base, FT has been implemented in recent years in several countries to support families where a parent has mental illness [e.g., the USA (Chicago), Costa Rica, Colombia, the Netherlands, Greece, Scandinavia (Norway, Sweden, Finland), Iceland, and Australia (Victoria)] (19). Internationally, there has been a growing trend, informed by the United Nations Convention on the Rights of the Child, to introduce legislation mandating support for children when a parent suffers from serious mental illness [e.g., the Swedish Health and Medical Service Act (20, 21)]. This legislation means that psychiatric services are obliged to take patient's children into consideration, including meeting their needs for information and support, and discussing issues of parenthood and the children's well-being (21). However, the continuing stigma around mental illness, especially as a parent, coupled with service provider constraints, often means that these policies are not implemented in practise (22).

Within the Irish context, whilst national practise guidelines have recently re-oriented toward a recovery,^1^ strengths-based model of care that recognises the needs of family carers and the value of family-focused mental health practise (23–25), there is no specific policy/practise guidance to support families with PMI in the RoI. Consequently, the national Health Service Executive (HSE) provided funding for the current research programme—called “PRIMERA” (**P**romoting **R**esearch and **I**nnovation in **M**ental h**E**alth se**R**vices for f**A**milies and children), the primary aims of which were to: (1) identify/develop, implement, and evaluate family-focused interventions for families with PMI; and (2) inform a “think family” care delivery agenda within mental health services in Ireland. Following an initial scoping study that demonstrated a lack of structured support for this population in the RoI, it was agreed with stakeholders that clinicians across 15 AMHS, CAMHS and child protection/welfare service sites would deliver Family Talk as part of a randomised controlled trial (RCT), with embedded qualitative and economic analyses (6, 26). FT was chosen for implementation as it: incorporates a structured “whole family” evidence-based approach; can be used with a range of mental disorders; provided freely available and high quality online training/resources^2^; and was replicable and capable of being implemented across sites in Ireland (6).

Despite the growing number of trial evaluations of FT, very few qualitative studies to date have investigated the experiences of families in attending FT. This means that little is known about the barriers and facilitators of change, intervention characteristics or contextual factors that may influence implementation and trial outcomes, particularly when delivered in real-world service settings (27). Indeed, the voices of service users, their families and particularly children, are rarely heard in controlled evaluations of FFPs (10, 28). Previously, it has been found that children may have a different perspective on “what helps” compared to parents and mental health practitioners (29). In addition, partners of service users have reported feeling uninvolved in research, which compounds their experience of feeling unsupported in their care burden by mental health services (30, 31). Thus, eliciting the views of children and other family members regarding FT delivery is important for informing the future development and refinement of this, and other similar, programmes.

Five qualitative studies eliciting family experiences of FT have been conducted, to date, all undertaken in Sweden, three within outpatient psychiatric settings (32–34), one within a substance misuse clinic (35), and another in an open care psychosis unit (36). With regard to the last of these, a companion study of clinician reports of family experiences of FT was also conducted (37). Table 1 summarises the participant characteristics across these studies). Collectively, the findings from these studies from both parents and children show that: the silence around mental illness in their home had been broken, they had greater understanding of mental illness, and more open family communication and closer relationships, although the level of improvements varied across and within families (32–36). Service-user parents felt more equipped and empowered in their parenting role and children expressed relief from fears, less monitoring of their parents, less carework in the home, and being able to spend more time with friends and other interests (33, 35, 37).

**Table 1:** Qualitative studies of Family Talk.

**References**	**Cohort interviewed**	**Recruitment agency**	**Parental diagnosis**	**Method & analysis**	**Country**
Pihkala et al. (32)	10 service-user parents (SUPs)	Adult psychiatry	Depression	Qualitative interviews, grounded theory	Sweden
Pihkala et al. (33)	14 children from 9 families, aged 6-17 yrs	General psychiatry	6 depression, 1 psychosis, 1 anxiety and ADHD, 1 with PTSD	Qualitative interviews, content analysis	Sweden
Pihkala et al. (34)	17 SUPs & 8 partners from 18 families	General psychiatry	11 depression, 2 personality disorder, 2 bipolar, 1 anxiety and ADHD, 1 psychosis and PTSD	Qualitative interviews, grounded theory	Sweden
Pihkala et al. (35)	7 SUPs, 7 partners & 10 children, aged 8-15 yrs	Clinic for substance use disorder	All 7 parents diagnosed with substance misuse comorbid with depression, anxiety and/or bipolar disorder.	Qualitative interviews, content analysis	Sweden
Strand and Meyersson (36)	8 SUPs & 7 children, aged 8-15 yrs	Open care psychosis units	4 schizophrenia and 4 schizoaffective disorder	Qualitative interviews, content analysis	Sweden
Strand and Rudolfsson (37)	11 Family Talk clinicians	Open care psychosis units	Parental psychosis	Qualitative interviews, thematic analysis	Sweden

*ADHD, attention deficit hyperactivity disorder; PTSD, post-traumatic stress disorder; SUP, Service-user parent*.

Arguably, these findings are potentially biassed in that they did not interview families who refused to attend or disengaged from the programme. High rates of refusal and attrition have been noted elsewhere, often due to competing needs for daily survival and fear of judgement (15, 37). A limited range of informants (e.g., mostly PMIs with depression, limited data from partners or those who disengage from FT), small sample sizes, and an overall lack of cultural diversity, underscore the need for qualitative analyses to be undertaken across a wider variety of settings and contexts. For instance, FT is not always delivered in countries with specific policy/practise guidance for this population.

This qualitative study was nested within an RCT of the Family Talk intervention in Ireland for families with parental mental illness and children aged 5–18 years; the aim of the RCT was to assess the nature and extent of any pre-post intervention changes in child and family psychosocial functioning (26) and data analysis is currently underway. The objectives of the current study were to: (1) investigate the experiences of families attending FT; and (2) explore the processes of change, contextual factors or intervention characteristics that may influence trial outcomes in mainstream mental health settings (26).

## Methods

### Participants and Settings

The larger RCT included 86 families (139 parents, 221 children) in 15 sites across the RoI, involving adult, child, and primary care mental health services, and Tusla child protection services (26). Families (parents and children aged 5–18 years) were recruited by clinicians in each site from their existing waiting lists, and written informed consent/assent was obtained for their participation in the research (26). FT was delivered in a mental health outpatient clinic and/or in the home by a mental health professional, typically a social care worker, social worker, or psychologist. Families were eligible where a parent had a formally diagnosed mental disorder, with 80% of service-users attending AMHS for various mental disorders and 20% receiving antidepressant medication or primary care psychological support under the governance of a General Practitioner (26). Due to the high risk of intergenerational transmission of mental disorders (2), and a desire among stakeholders to increase family-focused collaboration between traditionally segregated adult (AMHS) and child mental health services (CAMHS) (6), we included families where children attended CAMHS or primary care services for mental health issues, as well as families where children were not involved with mental health services (26).

**Figure 2:**
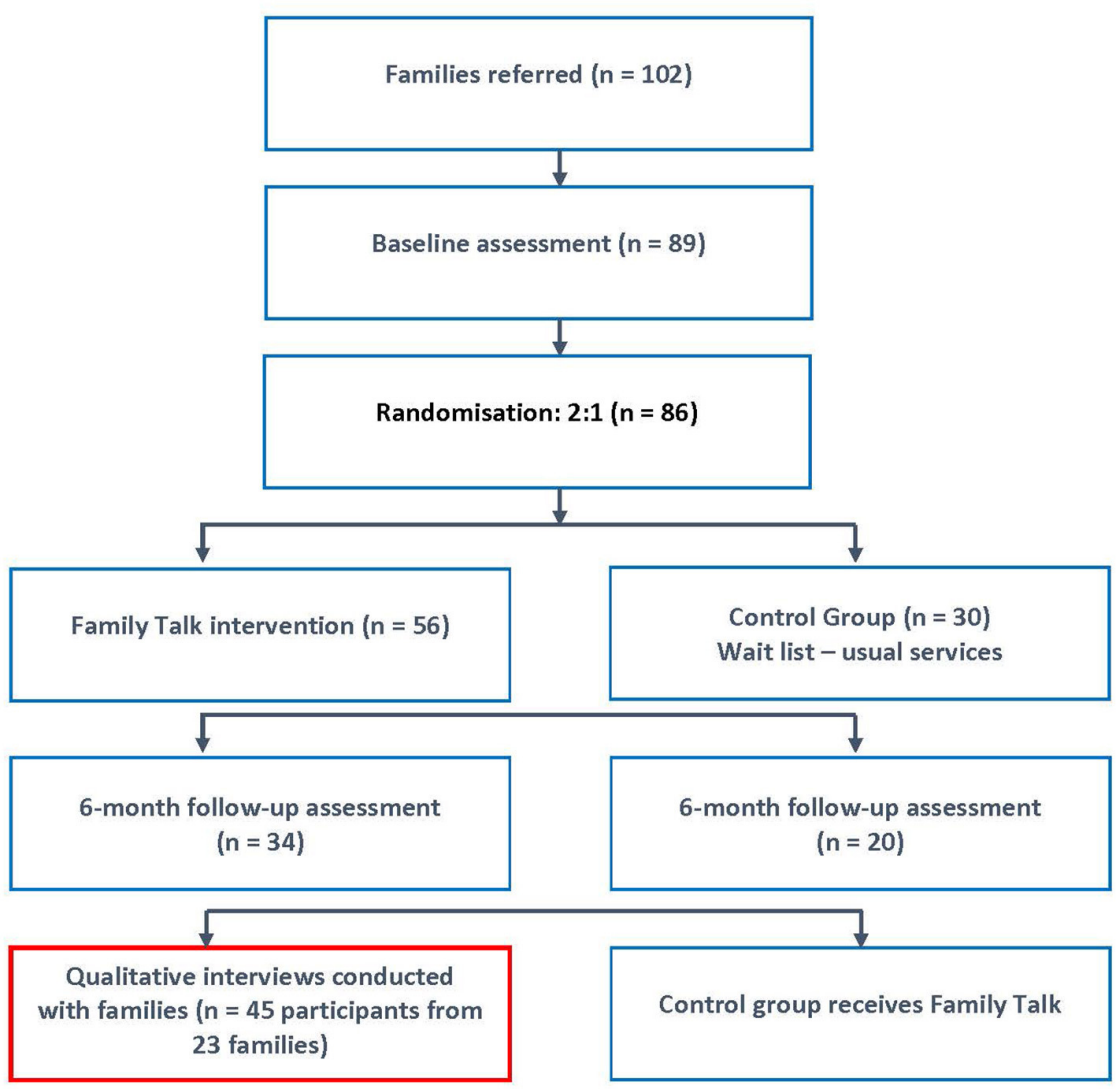
Study flow diagram.

Participants were block randomised, on a 2:1 ratio, to the FT intervention (*n* = 56) or to a treatment as usual control group (*n* = 30). Assessments were carried out at baseline and at six month follow-up periods. At six-month follow-up, attrition was 37%, the rate of which doubled due to the impact of the COVID-19 lockdown restrictions (22.8 vs. 45%). More details on study parameters can be seen in the study protocol (26). The flow of participants from recruitment through the RCT to the qualitative studies is shown in Figure 2.

For the qualitative study, a purposive sampling method was used to approach prospective participants (*n* = 34 families) for interview on the basis of key demographic variables (e.g., age, gender, lone parent, mental disorder, number of children, site location and (mainly socially deprived) socioeconomic status). A series of 37 one-to-one semi-structured interviews and 3 group-based family interviews were undertaken at 6 month follow-up with a total of 45 participants from 23 families, including 23 parents with mental illness (PMI), 7 partners and 15 children/young people aged 9 to 18 years. (While children over five could participate in FT, only those aged over 8 years could participate in the research process as the assessment measures were not suitable for the younger age group). Fourteen families attended all FT sessions while nine dropped out after completing less than three sessions, and were interviewed to provide a “negative case” analysis. In the RCT sample, mean attendance in the intervention group was 4.4 sessions (*SD* = 1.2), with 53% attending all sessions.

The qualitative sample had a largely similar profile to the larger RCT cohort in terms of the demographics indicated above. Twelve of the interviewed families were recruited by AMHS and eleven by CAMHS. Service-user parents (i.e., parent was attending mental health services, usually AMHS, for their mental health challenges) had a mean age of 41.6 years (*SD* = 8.2) and were predominantly female (18/23), Caucasian (22/23), and socially disadvantaged (15/23); approximately half (11/23) were lone parents and the largest proportion reported anxiety/depression (*n* = 14/23), followed by bipolar disorder (*n* = 4), Borderline Personality Disorder (*n* = 2) and psychosis (*n* = 3). Six of the seven partners who agreed to be interviewed were married. Ten of the 15 child participants were male with a mean age of 13.2 years (*SD* = 2.8), and approximately half (8/15) reported attending, or were on a wait list for, CAMHS. The children in the larger RCT tended to be more evenly distributed by gender and were also a little younger (*M* = 10.3; *SD* = 5.3), whilst 42% were attending either CAMHS or psychology/family services (Table 2). More details on the characteristics of interviewed families can be seen in Table 2.

**Table 2:** Characteristics of interviewed families.

**Family ID**	**Referring agency**	**PMI**	**Marital Status**	**Mental diagnosis**	**Age**	**Partner mental health**	**No. Children**	**Children's mental health/services**	**Interview configuration**	**FT attendance**
1	AMHS	Female	Living together	Schizophrenia	45	Substance use disorder & anxiety	5	One child has anxiety, attends family support service	Dyad with parents	Completed FT
2	AMHS	Female	Single parent	Anxiety	31	History of domestic violence	3	Son and daughter in CAMHS with ASD and anxiety/self harm	1:1 with PMI 1:1 with eldest son	Completed FT
3	AMHS	Male	Married	Bipolar	49	None identified	3	One son in CAMHS with ASD	1:1 with each family member	Completed FT
4	AMHS	Male	Married	Depression/ PTSD	38	None identified	3	Not in services. Eldest child on waitlist due to anxiety symptoms	1:1 with both parents & two children	Completed FT
5	AMHS	Male	Married	Low mood/ Anxiety	47	None identified	3	Not in services	1:1 with PMI	Completed FT
6	CAMHS	Female	Married	BPD	42	None identified	3	One child in CAMHS with self-harm & emotional deregulation	1:1 with each family member	Completed FT
7	AMHS	Female	Divorced	Schizophrenia	52	None identified	3	Not in services	1:1 with PMI	Left after 3 sessions
8	CAMHS	Female	Married	Depression	48	Depression symptoms	3	Eldest in CAMHS with depression and youngest with behavioural difficulties	1:1 with eldest child; group interview with other family members	Completed FT
9	AMHS	Female	Married	Depression	36	None identified	3	Eldest in CAMHS, feeling suicidal	1:1 with PMI	Completed FT
10	CAMHS	Female	Married	Anxiety/ Depression	40	None identified	2	Eldest in CAMHS with depression	1:1 with both parents & eldest child	Completed FT
11	CAMHS	Female	Widowed	Depression	37	N/A	3	Eldest in CAMHS, suicidal	1:1 with PMI	Did not start FT
12	AMHS	Male	Separated	Depression	43	N/A	1	Not in services	Dyad with father & son	Completed FT
13	CAMHS	Female	Single parent	Bipolar/ADHD	39	N/A	2	Eldest in CAMHS for stress/ADHD	1:1 with PMI	Completed FT
14	CAMHS	Male	Married	Depression	50	None identified	5	Three children in CAMHS – all with anxiety	1:1 with PMI	Completed FT
15	CAMHS	Female	Single parent	Depression	44	N/A	5	Two children in CAMHS – depression/anxiety and ADHD/ASD	1:1 with parent & youngest child	Completed FT
16	CAMHS	Female	Married	Depression	51	Depression & history of panic attacks	3	Not in services. One child on CAMHS waitlist, suicidal thoughts	1:1 with both parents	Completed FT
17	AMHS	Female	Lone parent	Psychotic depression	36	N/A	1	Not in services	1:1 with PMI	Did not start FT
18	AMHS	Female	Lone parent	Depression	48	N/A	2	Both children in CAMHS – social anxiety, self-harm	1:1 with PMI	Left after 2 sessions
19	CAMHS	Female	Married	BPD	35	None identified	4	One child in CAMHS with anxiety	1:1 with PMI	Left after 3 sessions
20	AMHS	Female	Married	Depression & anxiety	37	Depression & anxiety	2	Not in services	1:1 with PMI	Left after 1 session
21	CAMHS	Female	Lone parent	Bipolar	41	N/A	2	One child in CAMHS with anxiety	1:1 with PMI	Did not start FT
22	AMHS	Female	Lone parent	Bipolar affective disorder	42	N/A	2	Not in services but says child is depressed	1:1 with PMI	Did not start FT
23	CAMHS	Female	Lone parent	Anxiety	34	N/A	1	Child in CAMHS with social anxiety	1:1 with PMI	Left after 2 sessions

*ADHD, attention deficit hyperactivity disorder; AMHS, Adult Mental Health Services; ASD, autism spectrum isorder; BPD, Borderline Personality Disorder; CAMHS, Child and Adolescent Mental Health Services; FT, Family Talk; PMI, parent with mental illness; PTSD, Post traumatic Stress Disorder*.

### Data Collection and Analysis

Ethical approval (for both the RCT and qualitative study) was obtained from four research ethics committees including the HSE, the research institution where the research was carried out [name withheld for anonymous peer review], and two of the services with whom the research team worked (called Tusla and Saint John of God's Hospitaller Services).

Consent/assent forms were administered to parents and children, outlining details of the PRIMERA study, its potential benefits/risks, and where to seek help if necessary. Parents provided written informed consent for their children to participate and then their children provided written informed assent. Interview schedules were devised for each of the three participant groups in order to guide, and provide a framework for, interviews. These included questions such as “Tell me about your experience of FT, “What did you like about it?,” “What would you change about FT?” and “Would you recommend FT to other families?” Families who completed <3 sessions were asked their reasons for not completing FT. Interviews lasted between 15 and 40 min, with 33 (73%) conducted in participant's homes, and 12 (27%) via online platforms during the COVID-19 lockdown restrictions. There was some evidence of possible gatekeeping in three families with the PMI limiting access to family members. In addition, two parents requested to sit in on the children's interview. The interviews were conducted by experienced researchers [CM (*n* = 38), SMcGa (*n* = 7)], with lived experience of PMI, and with qualifications in psychology, mediation and psychotherapy. Given the stigma and impact of PMI, every effort was made to create a warm and non-judgemental atmosphere to ensure that participants felt understood. In addition, rapport had been established prior to the interviews as both researchers had prior contact with families during the baseline and 6 month RCT assessments (blinding was broken after the 6 month assessment to complete the qualitative interviews). Parents received a €25 gift voucher as a token of thanks for participating in the qualitative interview and children received a €10 voucher. Interviews were audio recorded with consent and transcribed verbatim.

The data from the interviews were analysed using constructivist grounded theory and MAXQDA software in order to identify and organise themes (38, 39). Analysis was also informed by the Medical Research Council guidance for complex interventions (27). Data were analysed using line-by-line and focused coding, constant comparison of codes to find similarities and variations within categories and hierarchical linking of categories to generate super-ordinate (or overarching) themes. The epistemological stance of constructivist grounded theory is more explicit than grounded theory in acknowledging the interpretive or constructivist nature of generating themes (38). The research interviewers were sensitised to honouring the lived experience of all participants (and particularly children) given the lack of data from this often invisible cohort but also due, in part, to their history of PMI. All interview transcripts were read by CM and MF, CM coded and analysed all of the data, while three authors (MF, SMcGa, SOC) independently assessed the reliability of coding on 12 of the 45 (27%) interviews. Reporting conforms to COREQ (Consolidated Criteria for Reporting Qualitative Research) guidelines (40).

## Results

Two overarching themes were identified from the analysis: (1) Benefits and experiences of FT and (2) Key barriers to participation (Table 3). A number of subthemes were also identified within each.

**Table 3:** Qualitative themes and subthemes of family experiences of Family Talk.

**Theme 1: Benefits and experience of FT**	
*From fear and silence to sharing and empowerment*	Experiences of service-user parents - Reduced stigma and worry - Deeper understanding of impact of MI on children - Better family relationships (communication, support) - Parental confidence and enhanced wellbeing
	Hearing the child's voice - Disclose hidden concerns and burdens - Better understanding of PMI - Relief and less worry - Warmer, more open family relationships
	Partners' experiences - Relief at having burden validated - Enhanced team approach to supporting PMI - Closer family relationships
*Facilitators of change*	Clinician skill
	Whole-family approach Timeliness/readiness
**Theme 2: Key barriers to participation**	
*Initial engagement phase*	Parental stigma and beliefs
	Lack of clarity for children on purpose of FT Service constraints
*Intervention phase*	Emotionally challenging, but in a good way
	Varied within-family experiences
	Covid complications Disengaging from FT
*Ending phase*	More child, family and follow-up sessions Need for additional supports

*FT, Family Talk; MI, Mental illness; PMI, Parental mental illness*.

### Theme 1. Benefits and Experiences of FT: From Fear and Silence to Sharing and Empowerment

Despite initial reluctance and fear about discussing mental health in a family context, the majority of families who attended FT (14/16) reported substantial benefits from participation, including: reduced worry and stigma, a greater understanding of the impact of PMI on family members; giving children a voice; improved parental confidence and support; improved family communication, problem solving, and warmer relationships. A total of four sub-themes were identified here.

#### Benefits to PMI

Three quarters of service-user parents (12/16) reported a reduction in shame, stigma and worry about being a “bad parent” following the intervention, which helped to improve their sense of well-being and parental confidence. Labelling was a common source of stigma. One service-user parent, for instance, agreed to participate only on the condition that the term “bipolar” was not used with his children. Another parent recalled the pejorative names used by his wife, such as “crazy” or “mentaller”. Such labelling encouraged the PMI not to share their suffering and to try to appear “normal.”

“*I became very good at hiding things, trying to adapt and fit in and mirroring other people that were deemed to be socially acceptable.”* (PMI 5)

“*After coming out and saying it to them, and talking to them about it, there is nothing to be ashamed of.”* (PMI 13)

“*It was hard. But it was very relieving because there was a lot of stuff that I would have been fearing to talk about or say out loud.”* (PMI 12)

FT also helped parents to have a better understanding of the impact of their mental illness on their children. While most parents feared that discussion of their mental illness would burden their children, they were relieved to learn that more open communication enabled them to better understand their child's perspective, and empowered them to address child concerns and unspoken inaccurate beliefs. For example, one son panicked if his mother mentioned the doctor or heard an ambulance siren, fearing she would be re-hospitalised. Another secretly feared his mother was dying from cancer, while children in another family felt that they were somehow to blame for their mother's illness. The FT sessions also allowed parents to explain frightening past behaviour to their children, thereby allaying anxieties. Listening to their children's accounts was an emotional experience for all parents who completed FT.

“*It helped us as a family to see from their [kids] vantage point how it affected them and try to give them what they wanted to try to move on.”* (PMI 4)

“*She [*daughter aged 11 years*] said she wasn't a very good daughter. When I was getting cross or why things weren't harmonious in the house, she felt that it was her fault.”* (PMI 16)

“*I was able to think of them more as people rather than my children that I'd be trying to protect, keep them safe from everything…Anything that they needed to talk about or worried about, and without fear of repercussion. Being able to say it in a safe place was good, for all of us really. There were tears and everyone at the end of it felt good and felt heard and respected in it.”* (PMI 13)

“*I just felt brilliant after it and I was able to tell them how proud I was of them and how much I love them. I can't just put words on it. But it has changed us for the better.”* (PMI 15)

The direct involvement of children also helped parents to re-evaluate their understanding of children's prior behaviour; instead of assuming that silence indicated the child's lack of awareness of the illness or lack of care for the parent, the PMIs realised that a child's silence is more often an attempt to protect the family and/or to avoid burdening them.

“*Beforehand I was saying, oh they don't want to talk to me… It's that they didn't want to be putting extra worry on me about anything because I had a mental health issue.”* (PMI 6)

“*I did not realise my eldest was being bullied for 2 years in school during my illness. He kept it to himself because at the time, he worried about me killing myself.”* (PMI 1)

Service-user parents also indicated that the sessions improved their communication with, and support from, their partners, as well as from their children. Overall, improved family interactions and relationships appeared to assist mental health recovery and personal and parental confidence

“*It gave me a sense of kind of, well not accomplishment…it was a huge sense of like, I'm doing this, I'm going to help* [the children]…*Having been through it, it gave me strength in a way.”* (PMI 2)

#### Hearing the Child's Voice

All but three the children (12/15) reported that they found FT to be helpful despite their initial reservations about attending and the emotional challenge of participating in individual/family session (Three of the younger children found it difficult to recall FT as they were interviewed 4 months following FT). Children indicated that they felt empowered by the opportunity to: voice “hidden” concerns about PMI, family dynamics, and other issues; and to develop a deeper understanding of their parent's mental illness. Previously undisclosed concerns included: distress with the PMI's behaviour (e.g., anger, social withdrawal, self-harm) and/or with arguments and tension at home, feeling overburdened by caretaking activities, being bullied, educational disruption, child depression, suicidal ideation, feelings of blame, fear, sadness and injustice/anger. Eldest and only children were more likely to carry a largely unacknowledged burden in caring for family members, especially during heightened presentation of symptoms. Caretaking responsibilities included: caregiving, cleaning, shopping, cooking, financial responsibilities, and looking after the emotional well-being of siblings and the other parent. The dominant unspoken message of silence around PMI, combined with an often unavailable partner (due to work pressures, absence from the home, and/or emotional disconnection), left children feeling overwhelmed and unsupported.

“*Dad was absolutely working his ass off to try and get money for us and taking care of Mam and running in and out of hospital. And doing school with us, trying to get us to do our homework and everything. He needed a lot of help. From a young age, myself and my sister had to take on a role, me more so because Dad was working and trying to provide. Mom was either in bed or in hospital, so I'd be like at school, have to come home, mind my siblings, my sister had to cook.”* (Eldest child, 16 years old)

“*I do get in a terrible mindset when it comes to my Mum's mental health because it's not nice… impacts on all of us as a family.”* (Eldest child, 18 years old)

“*I ask Mum for a lot of days off school because I get fed up with all the bullies...I used to have dark thoughts, not wanting to wake up.I do worry a lot. Because my Mum doesn't really have another person to help her, I'm normally that other person.”* (Middle child, 10 years old)

“*I pretty much have been in a really bad state since I was about nine, really low depression and suicidal. The only reason I didn't tell my parents was because my Mum's sick, my Dad doesn't care. It's not that they don't care, it's just that my Dad was working. I was kind of like, my Mum's in bed sick. I can't be talking to her, she needs to get better first.”* (Middle child, 14 years old)

Many children indicated that their parents were unaware how much they had been affected by tense/volatile home situations, and had hidden their concerns to avoid burdening parents. As parents became more cognisant of children's needs, family members were motivated to reduce levels of anger/arguments, and to relate to each other in more warm, caring and fun ways, thereby leading to reduced stress and worry and increased child well-being. For instance, family members made more efforts to connect with each other by having regular meals, spending time with each other, and being generally more cooperative and supportive. Siblings also advocated for each other's well-being in sessions, which helped to improve sibling relationships following FT. While there was still some evidence of parentification among children following FT, several expressed relief that FT had broken the silence, secrecy and stigma around mental illness within their families, and that as well as feeling that parents were now listening to them, they also experienced increased empathy and compassion for their parents whom they perceived as “trying their best” in challenging circumstances.

“*I found it was helpful for the family, like to talk about this, because usually when we're home, we don't really talk about it properly.”* (Youngest child, 12 years old)

“*I felt like the course has helped quite a lot to be honest. Family life has just got a lot easier. We're not arguing as much, we're not shouting. It's just easier to talk to people now...You have the resources to actually talk about it and try and sort it out.”* (Eldest child, 16 years old)

“*It was definitely worth doing. Because without the course we mightn't have known anything about it. And for him* [Dad] *to understand that we understand what he has. It's kind of improving him and us.”* (Middle child, 14 years old)

“*He* [FT clinician] *was asking how I was getting along with my Dad and my Mam, and I said, “Ok, we fight a lot, then it improved” and then we were called back in, and we were just saying, “Me and my Dad improved,” and he* [FT clinician] *said, “That's good.”* (Youngest child, 9 years old)

“*I think everyone's being a lot less aggressive, everyone's just trying to be a bit nicer to each other. I think everything's kind of been a lot calmer, especially with my Dad, he's been a lot calmer recently and he's started to kind of take other people's opinions and ideas into account.”* (Eldest child, 16 years old)

“*It helped me a lot. It made me feel better.”* (Eldest child, 10 years old)

#### Partners' Experiences

While partners corroborated the benefits noted above, the largest single gain from their perspective, was that FT provided them with a forum in which they could voice their experience of partnering and co-parenting with a PMI, often for the first time, despite their partner being in mental health services sometimes for up to 20 years. All partners spoke of the stresses of caregiving, financial and household responsibilities, feelings of loneliness, frustration and helplessness, and strains on their marital relationships. Partners indicated that their isolation was amplified by exclusion from the PMI's treatment/care plan, and that they lacked the knowledge or skills to help their partner. Maintaining the focus on the service user's ill health also negatively influenced some partners' self-care, with three partners (3/7) managing their own mental health difficulties including anxiety, depression and alcohol misuse.

“*You feel like you're carrying a whole house on your shoulders. You feel like a right tool. I can't do anything right. I can't say anything right. I can't help…I don't know what to do.”* (Partner 6)

“*I needed to ring someone, just for advice or help. But there was nobody.”* (Partner 4)

“*Over the last 4 or 5 years, we even talked about splitting up.”* (Partner 3)

All partners described relief at having their experiences validated by the FT clinician and acknowledged by the PMI. While heated discussions and angry outbursts were common in the initial sessions, they were seen as worthwhile as it increased understanding and empathy between parents on the burdens that each was carrying. Five partners indicated that their relationship with the PMI had improved following FT. Partners also expressed to the PMI that they wanted to know how best to support them and wanted to be involved in their careplans. The dialogical approach of FT sessions helped to encourage a team approach to supporting the PMI, helping both parents feel more connected.

“*It's an opportunity for him* [husband] *to hear me voicing the impact that it has on me in a very calm manner, because I'm in front of somebody else. It also takes away some of the guilt or the blame for me on his side… when you are more involved in the treatment.”* (Partner 3)

“*These sessions were great because we were both able to see where the other person was coming from.”* (Partner 1)

“*I think it [Family Talk] is 100% needed. As I said, there was nobody out there for me or the kids that I knew about… I can't compliment it enough. It's just the best thing that happened.”* (Partner 4)

#### Facilitators of Change

##### Clinician Skill

The majority of PMIs and partners indicated that it was the skill of FT clinicians that mediated the benefits for families. Parents welcomed the non-judgemental and strengths-based approach adopted by clinicians, and their skill in facilitating multiple perspectives across several developmental ages. In addition, the PMIs (12/16) appreciated the clinician-led, psycho-educational aspect of the programme, which led to a deeper understanding and normalisation of their mental health challenges.

“*Family Talk was very positive because there was somebody, a trained professional who had seen this before-it was in a way normalised. The kids were worried they were the only family in Ireland who had this problem.”* (PMI 5)

“*Everything that I talked about and went through, I had full support from her* [FT clinician]. *I can't even tell you how good she was. I can't say it enough. She was unbelievable.”* (PMI 12)

“*Everyone can say how they felt without any fear...Everyone felt very good afterwards and it was like a weight lifted… It's like a friendship with someone* [clinician] *that knows what you're talking about.”* (PMI 13)

##### Whole Family Approach

All family members believed that FT worked because it involved the whole family, and allowed multiple, often hidden, stigmas, concerns and burdens to be revealed and shared, thereby validating each person's lived experience, whilst also empowering them to be more supportive of each other. Participants indicated that the focus on the family unit had helped them to look beyond their individual burdens and to feel deeper understanding and empathy for each other.

“*It* [FT] *opened up the family and they talked about what they wanted to say and everything and you knew exactly where you stood, and it was up to you then to change the wrong things to try change them to the right things...It was brilliant because it brought out everything, the good and the bad, which was good.”* (Partner 7)

“*I just remember it was good for our family to actually talk properly without any kind of aggression, without any blame… everyone could just say how they saw things and people would put in their input without anyone kind of being upset about it. It was good to have like outside influences making sure everything was just calm.”* (Eldest child, 16 years old)

##### Timeliness/Readiness

Parents also indicated that timing, setting and their readiness for FT were important factors in engagement. If approached too early, they said that they might have denied the impact of their illness on their family/children. They also required a lead-in time to build up the courage and find words for the difficult initial conversations with their partners and children in order to convince them to participate. Furthermore, in order to engage properly with the programme, they indicated that they needed to have recovered from their worst symptoms. Parents also valued the flexibility of holding sessions within their homes or within clinics.

“*We did it during the summer and it was ideal, we walked to it* [clinic] *through the park and went for coffee afterwards, just the whole experience of going was great for the family.”* (PMI 4)

“*This time last year I wasn't feeling well so I was able to focus on it this time.”* (PMI 9)

### Theme 2: Key Barriers to Participation

**Figure 3:**
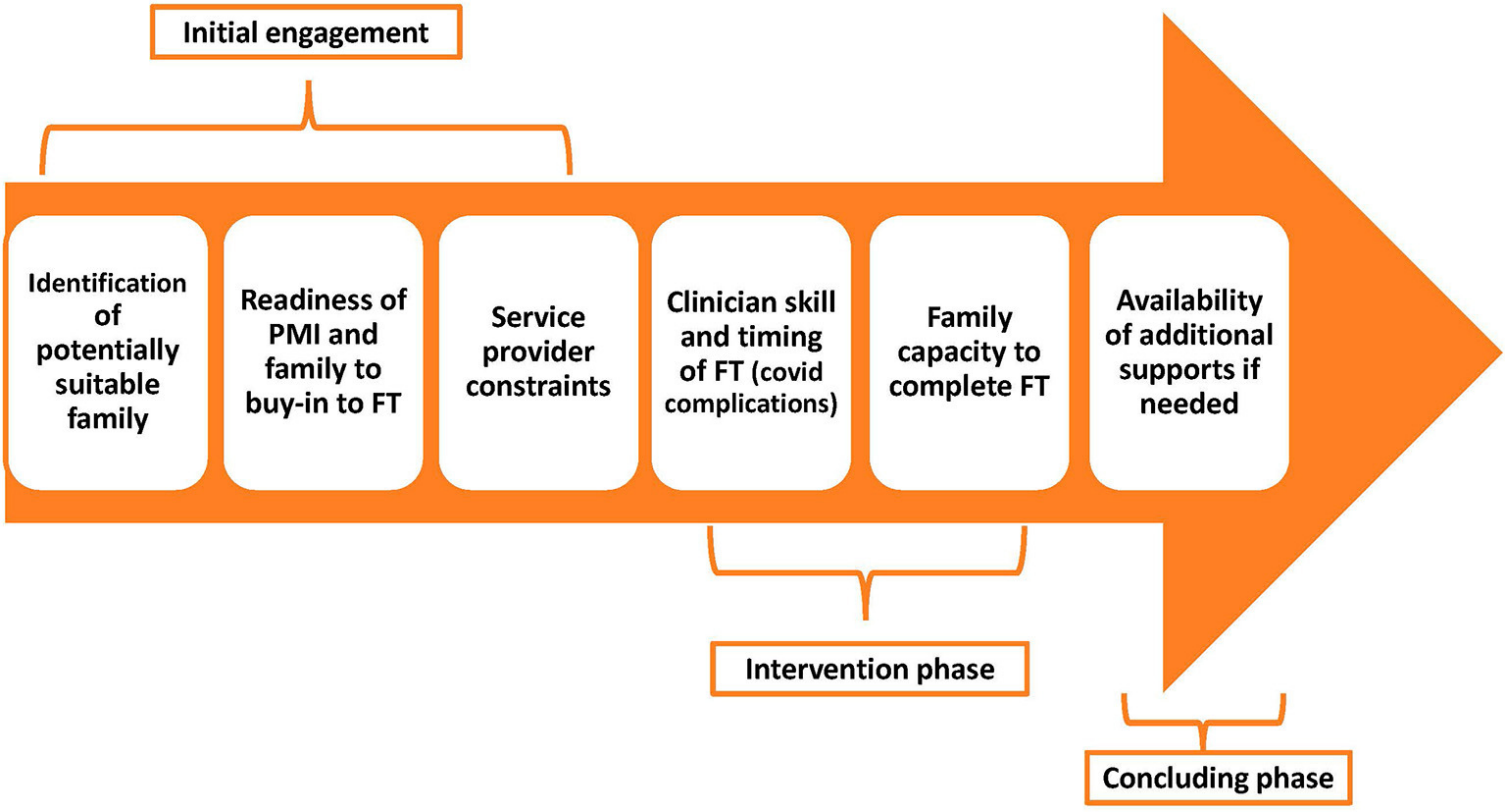
Challenges to participation in Family Talk during the engagement, intervention and ending phases.

Key barriers to participation occurred during one of three phases, each of which was identified here as a subtheme including: (a) initial engagement; (b) attending the intervention; and (c) the concluding phase. An overview of challenges to participation can be seen in Figure 3.

#### Initial Engagement Phase

The most common barrier for families attending FT was parental attitudes/beliefs and stigma around mental illness. Parents were hesitant about participating for the following reasons: they felt stigma and shame in openly discussing their mental health challenges in the family context; they believed they were protecting children from the impact of their illness by not discussing it; they feared what their children would say; and a few were not ready to acknowledge that their illness might affect their children. Families required extensive preparatory input from clinicians to allay their fears to persuade them to engage with FT.

“*The hardest part was actually admitting to him* [the child] *that I suffer with mental health problems. I hid away because for a long time, I have suffered with the stigma that goes with it.”* (PMI 15)

“*There's a very big stigma there. To make it easier to get through to him* [partner]*, I think having someone else outside* [clinician]. *Through that, I felt a lot safer to try Family Talk.”* (PMI 12)

Several children also highlighted their reservations about attending FT in terms of not understanding its purpose, fearing the focus would be on their wrongdoings (perceived or otherwise), and distrusting the viability of open discussion with typically uncommunicative parents. A couple of parents admitted that they had deliberately not clearly explained the purpose of FT to their children, for fear they would not attend.

“*I would have preferred a bit more information. I didn't know what to expect and when we went in to speak with the girl who was doing it, I didn't know if the questions were going to be directed at us or about our parents. I was getting agitated because I was confused.”* (Eldest child, 15 years old)

“*I wasn't clear what it was about so I didn't know what to say.”* (Middle child, 13 years old)

In addition, service constraints (e.g., staff turnover, uncovered maternity leave) disrupted/delayed FT delivery which meant that some families had an FT clinician with whom they were unfamiliar, meaning that additional time was needed to build rapport and trust before starting the intervention.

“*I had never met her* [FT clinician] *so I think if maybe we had one or two sessions before just to get her to know a little bit better.”* (Middle child, 17 years old)

Four of the interviewed families did not start FT due to family challenges and service constraint issues including: relapse in parental mental health; dealing with family crises (e.g., sexual assault, facing homelessness); deciding not to inform children about FT; being discharged from CAMHS before they could start FT; and suspension of FT delivery due to COVID-19 lockdown restrictions. It was interesting to note that discharge from CAMHS was cited as a reason for non-engagement because this reflects the lack of managerial priority given to family work and a perception by some CAMHS clinicians that working with parents is outside their service remit.

“*We really wanted to do Family Talk but our daughter told us she was sexually assaulted so it wasn't the right time.”* (PMI 20)

“*We were meant to go to it* [FT] *but then we were discharged* [from CAMHS] *and that was the end of it.”* (PMI 18)

#### Intervention Phase

The findings suggest that FT was challenging for many families despite the non-judgemental support provided by FT clinicians. Several parents/partners reported difficulties in speaking openly in sessions and/or listening to the experiences of family members, although most ultimately felt that it was worthwhile because it improved family communication. Partners and parents were shocked by their children's previously undisclosed revelations, what children had observed, and the internal narratives being used by children to make sense of the family situation (e.g., “Mum has cancer,” “I made Dad ill”). There was also some unease that clinicians might “selectively” reveal what children had disclosed in the child session, leaving parents feeling uninformed. Furthermore, a couple of service-user parents believed that their partners had undiagnosed mental illness, and consequently were unhappy that the focus was primarily on their own mental health challenges.

“*It was an eye opener. It was upsetting at the time, because to hear your child say certain things, it's really upsetting. But upsetting in a good way.”* (Partner 4)

“*We think we're protecting and sheltering them and, in the meantime, we're doing more damage.”* (Partner 6)

Similarly, most children (10/15) reported emotional challenges in engaging with FT. For instance, most children had no prior relationship with the FT clinician and some perceived them as asking too many personal questions too quickly, without allowing time for trust and rapport to build. Four children said that they felt shy and/or embarrassed when answering clinician's questions. A couple of children also indicated that they did not want to answer questions as their parent had not discussed their mental illness with them before the FT child session. Another child left the individual session and informed his mother that he had not revealed anything because the school encourages pupils “not to talk to strangers.” A few children found the family session emotionally volatile but ultimately helpful, while one teenager was initially upset in learning details of their parent's illness. Despite these challenges, the skill of clinicians in engaging children (e.g., allowing time to adjust, facilitating their voice being heard in the family session) had helped build a safe space for all but two to open-up and to engage with the process.

“*Sometimes she'd ask personal questions that I wouldn't feel comfortable answering...My mouth goes shut.”* (Middle child, 10 years old)

“*I was just sitting there quietly not really talking or saying anything. I felt completely thrown under the bus. I wasn't surprised. I would have liked more information.”* (Eldest, 16 years old)

“*It was kind of emotional but then it got helpful and pleasurable. We got to say what we wanted to say. Dad has become more open. He shows his emotions now. He used to bottle them up a lot before.”* (Only child, 12 years old)

Interestingly, within four families, there were widely varying experiences of the perceived utility of FT. In three families, both children and partners reported considerable benefits but the PMI did not. One PMI said that she “did not want to hear what others [in her family] were saying and blanked out,” whilst another was wary of discussing mental health with his children in terms of diagnostic labels and believed the intervention, particularly with the children, was of insufficient duration and should have been delivered years earlier. Both of these parents had severe/enduring mental illness, were currently feeling very unwell, and had a history of being unhappy with mental health service provision. In the fourth family, FT was delivered in the morning when the PMI was heavily medicated, thereby limiting her level of engagement. Furthermore, while the child reported many benefits, both the PMI and her partner found it less helpful. The PMI was disappointed that FT had not focused on her daughter's mental health difficulties or her husband's “control issues,” while her partner said he found it difficult to share his concerns as he believed it would aggravate his wife's emotional instability. These varied experiences highlight that each family member presents with a unique history and motivations and can present a range of challenges for clinicians when considering a family's readiness for FT.

“*Family Talk might not have helped Mum as much but it helped us.”* (Eldest, 18 years old)

“*I just don't think we got a whole lot from it. It is very one sided to be honest… when an issue did come up, if there was something with regards to myself or my husband, they just constantly kept bringing it back to “Well, how does that affect* [child]*?”* (PMI 10)

“[The PMI] *was getting so emotional because of her own opinions about things and stuff…I wasn't going to start dumping my own out there because it could have got messy and emotional. I didn't want to escalate any kind of like emotions. It was emotional enough. I was just kind of dealing with what was being brought up by* [partner] *and* [child]*.”* (Partner 1)

The COVID-19 pandemic restrictions have been shown to have had a considerable impact on population mental health and on those with pre-existing mental illness, both in Ireland and internationally (41–44). Seven families in this study were interviewed during the COVID-19 emergency, with three reporting sustained benefits from FT and that they were coping well with pandemic stresses, while four families reported increased mental distress and challenging child misbehaviour as a result of the restrictions; two of these families had disengaged from FT due to stigma/relapse issues and two indicated that FT delivery had been suspended due to the restrictions. Therefore, it appeared that the level of prior vulnerability and ability to engage with FT predicted how well families had coped with the stresses of the COVID-19 restrictions. In addition, one parent reported attending online sessions of FT for PMIs, partners, and older teenagers (16+) but these were not considered suitable for younger children or for family sessions and they had to wait until it could be delivered safely again in person and in line with COVID-guidelines.

“*I don't think we could have dealt with months of isolation if we hadn't done FT. We make time for each other now at this stage. We watch family films or to sit down for dinner, meals.”* (PMI 13)

“*It wasn't the same but we were able to talk with him [clinician] on zoom. It was a while before the children could be seen so it wasn't ideal.”* (PMI 5)

### Disengaging From FT

Families who disengaged from FT after three or fewer sessions (*n* = 5) gave the following reasons. One said that FT was too emotionally upsetting, with another feeling a sense of blame for causing her children's mental health issues. A number of other factors also contributed to disengagement including family crisis, relapse in symptoms, and having too many competing priorities. Additional delays/disruption in FT delivery due to the COVID-19 restrictions also led to some degree of disillusionment and disengagement from mental health services. This was more common in areas where mental health clinicians were partially redeployed to frontline COVID-19 duties and could only provide minimal telephone support to service users (41). Interestingly, those who disengaged from FT were almost twice as likely as “completers” to be lone parents (6/9 vs. 5/14)–suggesting that the stresses of lone parenting may also have been a barrier to engagement.

“*With covid, we are far less a priority for them. I don't know when or if we're ever going to get it.”* (PMI 22)

“*It felt like she was attacking me and it was my fault how the girls are...I don't need that.”* (PMI 19)

#### Concluding Phase

Despite benefiting from FT, most attendee families (*n* = 12) found the programme to be too short and expressed a desire for more child, family and follow-up sessions to build family communication. Families had high expectations of FT, which appeared to be linked to their need for more (often unavailable) psychological and family support from mental health services. Three families reported that they were referred to further mental health supports (e.g., dialectical behaviour therapy), while two others were given a list of alternative supports including national mental health charities. Most, typically, parents reported that FT clinicians provided closure by affirming their availability if future issues arose but the lack of follow-up was problematic for some partners who were not offered additional support and who were unable to pay for private treatment outside of statutory service provision. However, it is important to note that three attendee families were interviewed during the first COVID-19 lockdown which severely limited their access to mental health and community services and to other social/family supports at that time. Nonetheless, there were numerous indications that this population would likely benefit from longer-term (family-focused) mental health support.

“*It didn't feel like* [it] *was ready to be finished.”* (Partner 3)

“*I think more sessions with the family…and more time with the children would have really helped. The three of them went in one by one for 20 min. So it might have been a little bit rushed for them, they might not have had enough time.”* (PMI 16)

“*Family Talk is minimal… A taster...I think the hospital might have family therapy…But on a private basis so…”* (PMI 2)

### Discussion

This study is the first qualitative analysis of family experiences of FT conducted outside Sweden, the first situated within the context of an RCT of FT, and as part of the first nationwide endeavour to introduce FFP to adult and child mental health services in the RoI. The qualitative findings, in line with those reported in Swedish psychiatric settings (32–37), indicated many benefits for families who attended FT, including: reduced worry and stigma; a greater understanding of mental illness; giving children and partners a voice; improved parental confidence and family communication; and warmer relationships. Notably, the current study placed a greater emphasis on the caregiving, parental and financial stresses experienced by partners, all of whom for the first time had a forum in which to have their burdens validated, and a space in which to develop a more constructive team-based approach to supporting the PMI and the family unit. In addition, unlike previous qualitative studies, this study reported on the experiences of families who refused to attend or disengaged from the programme.

Importantly, the findings reported here, indicate that FT is acceptable and beneficial for families across different cultural/policy contexts, mental health settings, types of mental disorders, and among children with and without existing mental health challenges. Firstly, unlike Scandinavian countries where legislation has been introduced to safeguard children of PMI and where FT is implemented on a national level, the RoI lacks any “think family” policy/practise guidance for this population whilst service and public awareness of the need to support this population is erratic/unsystematic. There are also continuing high levels of mental health stigma in Ireland, which is an important barrier to help seeking (6, 45, 46). Given the challenges have been reported within Scandinavian (and other) countries in terms of translating family-focused legislation/policy into practise (22, 47), it was reassuring to find that FT was perceived as helpful by the vast majority of the families in this study, thereby highlighting the need for, and value of, such FFP supports for families where there is PMI.

Secondly, even though AMHS may appear the most natural fit for FT/FFP and provide a common context for the implementation of FFPs (45, 48, 49), families in this study reported benefits across both AMHS and CAMHS settings. FT is typically delivered as a preventive intervention to families whose children do not attend mental health services (14, 15) but in this study, most of the child participants, including those who attended CAMHS, reported that FT had improved their well-being and family relationships. Therefore, FFPs such as FT may also be helpful for children with existing mental health challenges, as well as promoting collaboration between adult and child mental health services, and increasing the identification of families through a “no wrong door” approach to family access, as promoted in the “Think Family” model in Northern Ireland (50).

Thirdly, there did not appear to be any notable variations across family experiences here in terms of the PMI diagnosis, thereby highlighting the suitability of FT for disorders beyond parental depression, which was the original focus of the programme (13, 14). Whilst a small number of adult service users who attended all FT sessions felt that it had not been helpful, their children/partners, and other service users with similar disorders, reported a range of benefits. Similarly, two PMIs, suffering from depression and Borderline Personality Disorder respectively, indicated that they disengaged from FT after two to three sessions as they felt blamed/upset by the idea that their mental illness may affect their children. Previous qualitative studies have indicated that while FT may work across a range of disorders, those with Borderline Personality Disorder or low-functioning psychosis are more likely to struggle with establishing a therapeutic alliance and/or exhibit a lack of understanding/insight into the impact of their mental illness on their children (34, 37). Low functioning service users may possibly require additional psycho-educational sessions and/or complementary groups for patients and children, in order to share experiences and learn about their mental illness and its impact on their children (37).

The findings reported here also highlight a number of important facilitators and barriers to engagement, which should help inform the future implementation of FT/FFPs and could be tested as mediators/moderators of RCT outcomes. Key facilitators included timeliness, clinician attributes and expertise, and involvement of the whole family. The clinician's role was key in: providing a setting for parent, partner and child voices to be heard and validated; normalising the family's lived experience; reducing fear and stigma through psycho-education; and teaching a strengths-based, problem-solving approach to improve family communication and interactions. Previous qualitative studies of FT likewise, highlight the value of clinician attributes of confidence, competence, warmth and non-judgmentalism in contributing to better family experiences (33, 34), whereas conversely, a perception that clinicians lack competency has been linked to disengagement (Unpublished data, Preventive Intervention Project, 2013, https://fampod.org/file.php/1/collaborations/Columbia_Application_of_PIP.pdf). An interesting study by Marston et al. showed that when parents were given a family-focused DVD, but did not receive the support of a clinician, their understanding of the impact of their illness improved, but they did have the confidence to open up a dialogue about the illness with their children (51). Thus, the presence of a trained clinician with appropriate attributes is a critical element of FT (and other FFPs) in terms of engaging families and potentially producing more positive outcomes.

Families particularly valued the whole family approach. As noted in previous qualitative studies, they felt that this had enabled all perspectives to be surfaced and heard, and improved mutual understanding and family relationships (32–34). Nevertheless, whole-family programmes appear to be less commonly delivered than parent-only models (48, 49), most probably due to the additional buy-in and logistics required in involving children. Our findings suggest that without the involvement of the child(ren), parents may not become aware of their worries and burdens as demonstrated here by the surprise and alarm that parents reported when their children opened up during the FT sessions. In addition, within parent-only interventions, parents may not be sufficiently supported to find the right words to have a conversation with their children, and may also lack a forum to realise just how much their children/partners want to support them, but have been prevented from so doing by the silence and stigma associated with discussing mental illness within the home. Two RCTs which have compared FT with the parent-only *Let's Talk about the Children* found FT to be more effective in reducing child emotional symptoms and improving the parent–child relationship (13, 15).

Similarly, child programmes that exclude parents may reduce the likelihood of mutual understanding as parents lack the opportunity to discuss their stigma concerns, to gain insight into the impact of their mental illness on their children, and/or to build parental confidence. Enhancing parental confidence and competence has been identified as key to shaping the quality of parent-child relationships (52). Significantly, the involvement of partners is less common in the implementation of FFPs (3). The findings from the current study indicated that FT provided a forum, usually for the first time, for partners to express their burdens and stresses and to communicate with the PMI about how they can better support them. Given the level of burden and stress reported, and the protective boundary provided by a healthy second parent (2), it is imperative that services/FFPs help to strengthen the “safety net” that co-parents provide in families with PMI. Recent filicide tragedies in Ireland (e.g., McGinley case) highlight the ultimate cost of not involving family members in the service user's treatment (53).

Significant barriers to participation were also reported in the current study. Most family members, including both completers and drop-outs, indicated that they had experienced challenges in engaging with FT in the initial phase. Parental fears and stigma around mental illness were the most commonly reported barriers to participation. Children also expressed reservations about attending, indicating that they were uninformed about the purpose of FT and wanted prior contact with the clinician prior to commencing FT sessions. Other family barriers to engagement were also noted, including relapse in symptoms and family crises. Previous qualitative studies have similarly highlighted that fear of judgement and/or competing needs for daily survival may impede family engagement (32, 37). These findings suggest that clinicians may benefit from the development of FT/FFP training videos/protocols to promote effective engagement strategies and address potential barriers to participation and retention. For instance, addressing issues of stigma, readiness/timeliness, consent and confidentiality during the recruitment process and including quotes/videos from previous FT attendees may help to improve engagement (54). In addition, a child-friendly recruitment approach that used age-appropriate marketing literature and involved a meet-and-greet session with the facilitating clinician might help to address children's concerns about attending. Lastly, low functioning PMIs may benefit from additional psycho-educational sessions and complementary group supports to promote engagement (37).

Service constraints were also an inhibitive factor in family engagement with the programme. The capacity of FT clinicians to build rapport and familiarity with the family beforehand was undermined by high turnover of personnel and under-resourced mental health teams. In addition, a small number of families were discharged from AMHS/CAMHS before they could start FT, while several other families disengaged due to their unhappiness with long waiting lists, and delays/disruptions due to the COVID-19 lockdown restrictions. These difficulties reflect general underfunding of mental health services in the RoI, alongside a lack of policy/practise priority given to supporting this population in an Irish context (26, 45, 55).

Some challenges were also noted during the intervention phase. Firstly, while most families reported that FT was ultimately worthwhile, it was also seen as emotionally challenging at times. Many reported difficulties in speaking openly in sessions and/or listening to other's experiences and indeed, this was also shown in research by Pihkala et al. (33) in Sweden. The clinician's skill in facilitating multiple perspectives was instrumental in ensuring that family members could listen to each other without becoming overly defensive or upset. Secondly, there was some evidence that children within two families did not receive sufficient time in their individual child session (e.g., 20 min each). Moreover, while children largely reported benefits from FT, there was little mention of fun within sessions. Therefore, children may benefit from the inclusion of some light relief at the beginning or close of sessions (e.g., ice breakers, child-friendly videos, closing “fun” take-home exercise), as used in, for example, the Kidstime intervention (56). Thirdly, the COVID-19 lockdown restrictions had implications for the delivery of FT, including blended adaptation (both in-person and online sessions), as well as family disengagement following repeated delays to delivery. Notably, there was considerable variation in the capacity of sites to deliver FT during the lockdowns with some mental health staff partially redeployed to frontline COVID-19 duties and providing minimal phone support to patients while clinicians in other areas were able to continue home visits and outpatient clinics, following COVID-19 guidelines (41). Reassuringly however, it is likely that the future implementation of FT will be conducted in person in view of the >90% uptake of vaccination in the RoI (57).

Lastly, while most families benefitted from FT, they indicated a desire for additional child, family and follow-up sessions, thereby suggesting that some of their needs had not been met. This was also noted by FT clinicians working with lower functioning psychosis patients in Sweden (37), although it was not reported by families experiencing depression (32–35). Where possible, the concluding phase of FT should signpost families to additional family and mental health supports as required. Given the complexity of service user needs, a flexible spectrum of family-focused services may be necessary, as demonstrated internationally (58), although this level of family resources is not currently available in Ireland (6).

#### Strengths and Limitations of the Study

This study is the first qualitative analysis of family experiences of FT conducted outside Sweden and the first conducted within the context of an RCT and national programme to introduce FFP for families with PMI, in this case within the RoI. Service-user parents are typical of those who take part in RCTs and qualitative studies of FFPs (28), but the current study involved the recruitment of a large and diverse sample (in qualitative terms) of both child and adult participants, including PMIs, partners, children, and “drop-out” families. In addition, our sample was recruited from a number of mainstream adult and child mental health services and encompassed a variety of mental disorders as well as including both children who were and were not attending CAMHS. The analysis yields further important insights into the barriers and facilitators of implementation, as perceived by families, and will help to support and amplify the clinician experiences of FT which are reported here in a companion paper (as well as the RCT results when they become available).

The study was limited in a number of ways, including firstly, the transferability of the findings across different cultural contexts. However, the description of the study context should help in this regard. It is also possible that the findings may be biassed in that families who agreed to be interviewed had a more positive experience of FT, and in a small number of instances, we believe that gatekeeping from the PMI may have potentially excluded feedback from other family members who were invited to participate in the research. Importantly though, we interviewed nine families who had disengaged from FT after three or fewer sessions as well as families who had completed FT. In addition, the interval between FT and the child interviews (ranging from 3 to 5 months) created recall difficulties for three of the younger children in our sample, although the remainder (*n* = 12) had much to say about their involvement. This interval was necessary due to blinding in the RCT which had to be retained until after the 6 month assessment had been completed. Lastly, 7 of the 23 families were interviewed during the first COVID-19 lockdown, which severely restricted access to services and led to increased levels of psychological distress in the general population in Ireland (42), both of which may have impacted their experiences and views.

#### Implications for Policy, Practise and Research

Our findings highlight the value of a whole family approach when a parent has mental health challenges, particularly in revealing the hidden burdens that children carry, reducing fears and stigma, and improving empathy and communication among parents and children. The findings illustrate that FT can be successfully implemented across adult and child mental health settings and with families experiencing different mental disorders, thereby reflecting, at least to some extent, a “no wrong door” approach to identifying and supporting families. Key facilitators to implementation included delivery by a competent, non-judgmental clinician and family readiness to participate. The primary barrier to FT implementation was recruiting and engaging with families in the initial phase due, in large part, to family challenges and service constraints. Engagement may be improved if clinicians address issues of stigma, readiness, consent and confidentiality during the recruitment process and use quotes/videos from previous FT attendees. In addition, children's concerns about attending could be addressed using age-appropriate marketing literature and an initial meet-and-greet session with the facilitating clinician.

Our findings also suggest that FT may not be suitable/sufficient for all families (e.g., low functioning service users) and should, ideally, be implemented as part of a suite of lower and higher intensity FFPs (58). There is an urgent need in the context of the RoI, to introduce “think family” practise guidelines and to provide dedicated funding to develop a multi-level, public-health response to identifying and supporting these families, as has been done in, for example, Scandinavia and Australia (33, 47). Under the United Nations Convention on the Rights of the Child (20), children have a right to a childhood and not be used as unpaid/unsupported carers filling gaps in service provision. Moreover, when child welfare is not considered in the treatment of service-user parents, it increases their risk of developing mental disorders and becoming the next generation of service users, and, in the most tragic (but thankfully rare) cases, can lead to their death by filicide (53). Internationally, systemic barriers to change need to be addressed, including mandatory auditing of the parenting status of adult mental health users, balancing the priority given to patient confidentiality with unmet family needs, increased collaboration between traditionally segregated AMHS and CAMHS services, and equipping clinicians with time and resources to undertake FFP.

Further qualitative and quantitative research on family and clinician experiences of FT implementation is required across different cultural/policy contexts, mental health and family settings, types of mental disorders and level of child mental health difficulties. Further research is also needed on the types of families that are more likely not to engage with FT, and to identify measures and/or supports that might increase engagement. For instance, there may be value in developing and evaluating training videos that teach recruitment strategies to see whether they improve engagement. In addition, qualitative analyses may inform RCT evaluations of FT/FFPs; for instance, RCTs could include as outcome measures, benefits identified in qualitative analyses, such as reduction in stigma, parental confidence/competence, service-user mental health, partner well-being, and family functioning. Moreover, facilitators and barriers to implementation identified in qualitative studies could be tested as moderator/mediator variables in quantitative research.

## Data Availability Statement

The datasets presented in this article are not readily available because where participants provided consent/assent, an anonymised version of their data will be stored in the Irish Qualitative Data Archive. Ethical approval from an approved higher level institution will be required if the data is to be used in future research. Requests to access the datasets should be directed to Mairead.Furlong@mu.ie.

## Ethics Statement

The studies involving human participants were reviewed and approved by four ethics committees: the Social Research Ethics Committee in Maynooth University, Ireland (Reference number SRESC- 2018-100), the HSE Research Ethics Committee, Tusla Ethics Review Committee, and the Saint John of God's Research Ethics Committee. Parents/legal guardians provided written informed consent for their children to participate in the study, followed by their children providing written informed assent to participate.

## Author Contributions

CM, MF, and SMcGi conceived and designed the study. CM conducted interviews and coded transcripts, with 25% of transcripts independently coded by MF, SMcGa, and SO'C. CM prepared the initial draft, with subsequent drafts undertaken by MF, with input from SMcGi, CM, and SMcGa. All authors contributed to the article and approved the submitted version

## Funding

The PRIMERA research programme is funded by the Health Service Executive and the Maynooth University Higher Education Authority COVID-19 Costed Extension Fund.

## Conflict of Interest

The authors declare that the research was conducted in the absence of any commercial or financial relationships that could be construed as a potential conflict of interest.

## Publisher's Note

All claims expressed in this article are solely those of the authors and do not necessarily represent those of their affiliated organizations, or those of the publisher, the editors and the reviewers. Any product that may be evaluated in this article, or claim that may be made by its manufacturer, is not guaranteed or endorsed by the publisher.

## Footnotes

^1^The approach argues against just treating or managing symptoms but focusing on building the resilience of people with mental illness and a change in outlook that is related to leading a meaningful, purposeful life, with or without ongoing episodes of illness (23).

^2^ https://emergingminds.com.au/online-course/family-focus

## Abbreviations

ADHD: attention deficit hyperactivity disorder
AMHS: adult mental health services
ASD: autism spectrum disorder
CAMHS: child and adolescent mental health services
FFP: family-focused practise/programmes
FT: Family Talk
HSE: Health Service Executive
MI: Mental illness
PMI: parents with mental illness
PRIMERA: **P**romoting **R**esearch and **I**nnovation in **M**ental h**E**alth se**R**vices for f**A**milies and children
PTSD: post-traumatic stress disorder
RCT: randomised controlled trial
RoI: Republic of Ireland
SUP: Service-user parent.
